# A morphometric analysis of *Tobleriabicuspis*, a Voltziales seed cone from the early Permian Jambi palaeoflora, Sumatra (Indonesia)

**DOI:** 10.3897/phytokeys.119.29555

**Published:** 2019-03-25

**Authors:** Isabel M. Van Waveren

**Affiliations:** 1 Naturalis Biodiversity Center, Postbus 9517, 2300 RA, Leiden, the Netherlands Naturalis Biodiversity Center Leiden Netherlands

**Keywords:** Asselian, Cathaysia, mesic-xeric, early conifer cones

## Abstract

*Tobleriabicuspis*, a coniferophyte seed cone, is described from the Jambi Palaeoflora, Sumatra of Asselian (early Permian) age. A morphometric analysis based on cones, paired fertile units, and fertile and sterile scales, demonstrates their close relationship. Small paired fertile units occur mainly in cones. Medium-sized paired fertile units occur mainly on scales. And large paired fertile units are mainly dispersed. The cones are considered female and the paired fertile units are considered to represent the seeds. The cones are composed of helicoidal, bilaterally symmetrical and deeply incised scales with paired seeds. A comparison can be made with the Voltziales female taxon *Schizolepis* from the Triassic and Jurassic. *Tobleria* is regarded as having a voltzian Voltziales affinity and dates from approximately 16 to 26 million years before any other such cones.

## Introduction

The evolutionary trend from early to late conifers is that of a radial fertile shoot in the axil of a leaf to a fertile scale fused to, or free from, a subtending bract (Florin 1951, Meyen 1997). Radial fertile shoots (walchian Voltziales) occur in late Palaeozoic taxa, while scales with sessile seeds and a (partly) fused subtending bract typify the Mesozoic and Cenozoic conifers (Anderson et al. 2007). The transition between the two took place in the voltzian conifers during the late Palaeozoic. Here the informal group of the walchian Voltziales is typified by radial shoots while the informal group of the voltzian Voltziales is defined by a fertile scale carrying sessile seeds (Rothwell et al. 2005). More precisely, the proliferous Pennsylvanian Hamilton Quarry produces chiefly walchian Voltziales (Anderson et al. 2007) while the Majonicaceae, belonging to the voltzian Voltziales (Rothwell et al. 2005), have been demonstrated to occur in the late Early Permian, thus indicating that evolution of the scale architecture and diversification among conifers took place before that period (Looy 2007).

Jongmans and Gothan (1925, 1935) described co-occurring organs from the early Permian Jambi palaeoflora, which they indicated as ‘seeds’ and scales. As these seeds and scales are often found superimposed, Jongmans and Gothan (1925) considered them to represent one functional organ, which they called *Tobleriabicuspis*. Van Waveren et al. (2007) reviewing the Early Permian Jambi palaeoflora, and noting three cones in the collection found in association with *Tobleriabicuspis* suggested they were related, and considered a conifer affinity for the scales, seeds and cones. As the early conifer transition between walchian and voltzian Voltziales took place between the Pennsylvanian and the Early Permian, the voltzian or walchian affinity of *Tobleriabicuspis*, appearing between the two in the Asselian, is of interest and requires verification.

A detailed morphometric analysis of the fertile units and scales, dispersed, superimposed and inside the cones, is presented. The statistical treatment of the fertile unit, and scale width and length, is used to demonstrate the coherence of the cones and the dispersed material. Details of the fertile units, scales and cones are presented to support an interpretation of *Tobleriabicuspis* as a seed cone displaying features indicating a voltzian Voltziales affinity.

## Material and methods

### Geographical and geological context

The Jambi palaeoflora is composed of impressions and coalified compressions that are kept at Naturalis, the Netherlands Center for Biodiversity. The samples are small in size (commonly 100 mm long, but sometimes up to 400 mm). Some cuticles could be retrieved from the samples, but no diagnostic features were observed.

**Figure 1.**
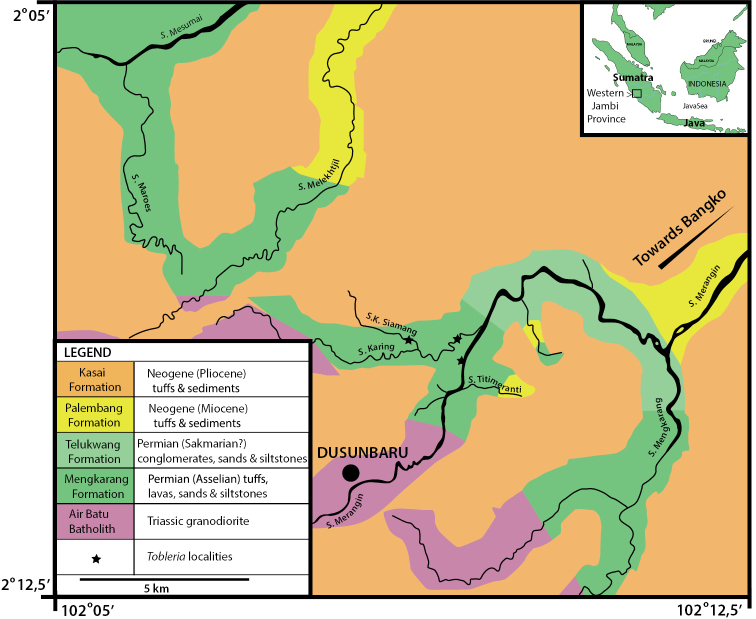
Map of the Bangko area showing the outcrops of the Mengkarang Formation along the distributaries of the Merangin River where *Tobleriabicuspis* was found.

The samples originated from outcrops along several rivers and their tributaries. These rivers cut into silicified Permian volcaniclastic rocks from the westerly mountainous region of the province of Jambi (Zwierzycki 1935, Tobler 1917, 1922). *Tobleriabicuspis* was found in localities situated along tributaries of the Merangin, namely the Karing and the Ketidoeran Siamang (see Fig. 1). These localities belong to the Mengkarang Formation (Suwarna et al. 1998).

The 500 m thick Mengkarang Formation is represented by eight fining-upwards intervals, each formed at the base of the formation by a basal pyroclastic accretion wedge composed of tuffs, and overlain by tuffaceous sandstones, organic shales and limestones, while at its top the tuffaceous sandstones are more common and basalts form the base of the intervals (Van Waveren et al. 2018). Fusulinids from the Merangin section suggest a late Asselian age (Vachard 1989) and, lately, isotopic age evaluation has indicated an Asselian age between 296.77 and 296.14 million years (Van Waveren et al. 2018). The Mengkarang Formation is overlain by the Telukwang Formation that is characterized by thick polymict conglomerates alternating with tuffaceous sandstones and shales (Suwarna et al. 1998). The Mengkarang and the Telukwang formations are intruded by the Triassic Air Batu granodioritic batholith (Zwierzycki 1935). The Miocene Palembang Formation, consisting of shales, sandstones and coals, is found discordantly on the Permian (Zwierzycki 1935). The Pliocene Kasai Formation, consisting of more than 400 m of tuffs, tuffaceous sandstones and siltstones, tuff breccia, lignites and peats (Suwarna et al. 1998), in turn, overlays the Palembang Formation discordantly (Zwierzycki 1935). *Tobleria* occurs in the upper half of the Mengkarang Formation. These occurrences and their mesic-xeric palaeoecological context were recently described in Van Waveren et al. (2018).

Palaeomagnetic results indicate that the Mengkarang Formation, in which the *Tobleria* remains were found, was positioned approximately fifteen degrees north of the equator (Suwarna et al. 2000). The presence of reefs and the absence of growth rings in wood (Van Waveren et al. 2005, Crippa et al. 2014) support the palaeomagnetic results, indicating that the Jambi flora grew in a tropical environment. The petrography of the section indicates that the Jambi palaeoflora was caught in the tuffs, pyroclastic flows and gravity flows of an active volcano from a volcanic arc where fluvially reworked tuffs and ashes form an apron to the more central volcaniclastic deposits (Donovan et al. 2013, Matysova et al. 2017, Van Waveren et al. 2018).

## Material

A total of 17 samples from the 1925 Jambi collection held *Tobleriabicuspis* organs. These organs consisted of cones, fertile units and scales, fertile and barren. Fertile units were defined as paired, closed, darker circular rims or paired, dark, disc-like areas, respectively. Scales were considered to be bilateral symmetrical, sub-rounded, darker areas with two apices. The possible nature of these barren scales as bracts is fully developed in the discussion. Scales were found in all 17 samples (old collection: 45308-20, 45468, 45471, 45617; and 2006 collection: KS 28). All samples from the 1925 collection had comparably fine-grained lithologies. The sample found in 2006 along the Ketidoeran Siamang stream is composed of light grey medium – to fine-grained tuffaceous sandstones and was collected from a bed of 30 to 40 cm in thickness that was formed by a volcanic mass flow. The remaining samples found in 1925 were also collected from that stream and have the same lithology. Three samples (45468, 45471 and 45311) were collected along the Karing River. These samples consist of light beige, finely banded, tuffaceous mud – and siltstone. They comprise the cones. One of the cones is under an angle to the bedding, indicating that it fell into the soft mud that kept seeds, scales and cones in the same vicinity. A number of coarser-grained tuffs from the old Naturalis Jambi collection did hold relatively large, faintly bifid scales, but preservation was too poor for any type of data acquisition.

**Figure 2.**
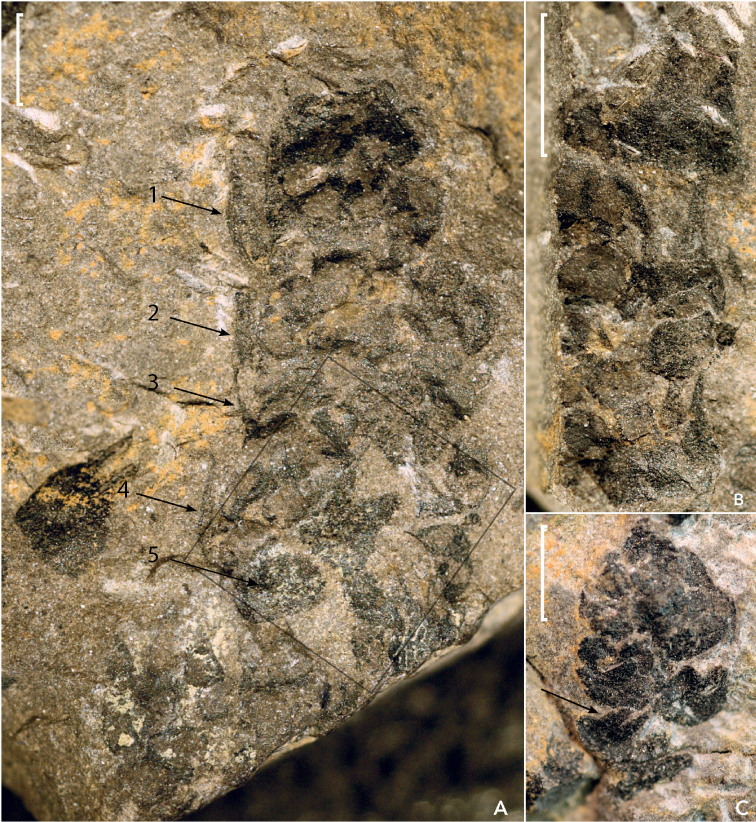
The three cones from sample 45311: **A** cone C1 from 45311 Aa, the black frame indicates the area that is detailed in Figure 5**B***Tobleriabicuspis* right edge of cone C2 fragment showing bicuspid scale and seeds **C***Tobleriabicuspis* cone C3 fragment showing bicuspid scale. Arrows 1–4 in Figure A indicate scales in side view, arrow 5 indicates the cone axis. The arrow in Figure C indicates a bifid scale. Scale bars: 10 mm (**A**); 2,5 mm (**B**); 5 mm (**C**).

**Figure 3.**
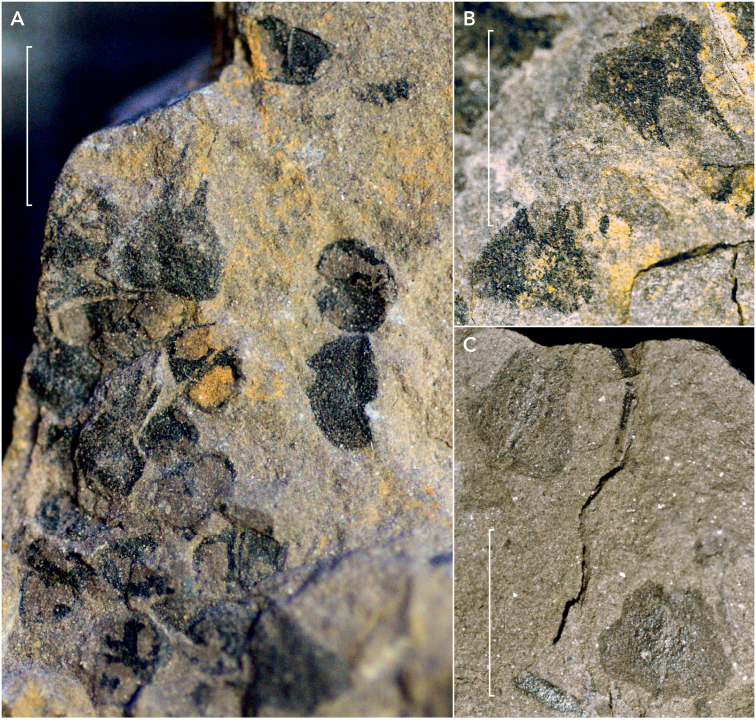
Associated seeds and scales **A** Dispersed seeds and scales (sample 45311a) **B** Scale with hart shaped scar and scale with darker central area (sample 45471) **C** Scale seen from adaxial side showing striation pattern and scale seen from adaxial side showing seed remains (sample 45313). Scale bars: 5 mm.

Sample 45311 consisted of five fragments that could be reassembled into a single specimen (Aa, Ab, B, C, D). Two cones were observed in fragment 45311A, both on part (Aa) and counterpart (Ab). The cone (C1) positioned centrally is largest and its structure can best be described using the Aa side (Fig. 2A). The second cone (C2) is positioned on Aa left of the central cone along the edge and only the right side of that cone is visible (Fig. 2B). On the counterpart Ab, C2 is observed to the right of the central cone. A third cone C3 in sample B is indicated by an apical portion (Fig. 2C). Fragments B and C (Fig. 3A and B) have both fertile units and bifid scales, with or without the fertile units. Fragment D only has a *Pecopterishemitelioides* fragment.

Preparation of cone C1 on sample Aa allowed the visualization of its apex that was buried a bit deeper in the shale, thus indicating that the cone was positioned under at an angle to the bedding before compaction. A scale apex was also prepared on the left apical border of Cone 1 (sample Aa). Dispersed fertile scales and fertile units were found in seven samples (45308, 45310, 45311, 45313, 45320, 45468 and KS 28) (Fig. 3A–C).

### Quantitative and statistical analysis

**Figure 4.**
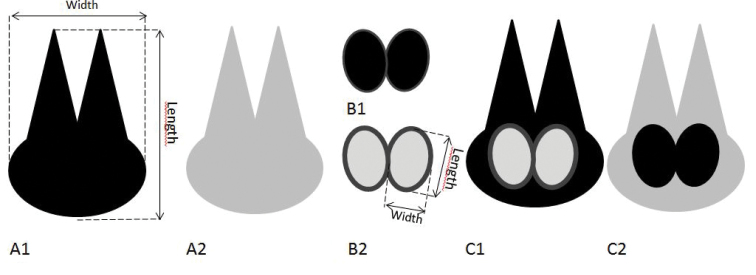
Diagram showing empty scale, opaque (**A1**) or lighter (**A2**), fertile units as dark discs (**B1**) or dark circular rims (**B2**), and fertile scale with lighter fertile units on darker scale (**C1**) or darker fertile units on lighter scale (**C2**) (see also Figure 6). It is indicated how length and width of scale and fertile unit are measured.

Pictures and measurements were made with a multi-stack Zeiss SteREO Discovery V20 microscope with a Zeiss AxioCam MRc 5 for photography, and the associated program AxioVision. Measurements were preserved on the pictures and data were exported into an Excel file. The lengths and widths of the fertile units (Fig. 4A) were measured while noting if they were free (dispersed), on a scale or in a cone. The length and width of the scales (Fig. 4) was measured while it was noted if they were dispersed, empty, fertile or in a cone. Two proxies were used for the size (length x width) of the scale (1) and fertile unit (2).

For all the data handling, the program PAST (PAleontological STatistics) version 2.12 (Hammer et al. 2001) was used. To analyze the data distribution NIST/SEMATECH e-Handbook of Statistical Methods (http://www.itl.nist.gov/div898/handbook/, 29/11/2017) was used. The degree of skewness was given in Excel.

### Fluorescence analysis

As the samples were too large for a fluorescence microscope, they were analyzed with the Diamondview. The instrument illuminates the surface of the sample with intense ultraviolet light specially filtered such that almost all of the light reaching the sample is of wavelengths shorter than 230 nm. To examine a sample, it is inserted into the stone holder from the port at the front of the unit. The stone is first exposed to visible light in order to focus the camera to the area of interest, after which it is illuminated with ultraviolet light and the fluorescence image is recorded to be exported to the computer (Welbourn et al. 1996).

## Descriptions

### Cones

All three samples displayed compact cones with densely packed scales. The most complete cone fragment C1 has the apical portion preserved and is positioned on the central part of sample 45311 Aa and 45311 Ab. It is 24 mm long and 8–9 mm wide at the base, representing its broadest point, and tapering apically to a width of 4–5 mm at 2 mm from the apex. The rock sample was broken at the base of the cone and the length of the intact cone remains unknown (Fig. 2A).

Fertile units and scales in cones C1 and C2, on sample 45311 Aa and Ab, were too numerous to each be rendered photographically, but were all measured and are treated (below) in the statistical analysis. On cone C1 (Fig. 2A) the fertile unit size decreases slightly in the apical direction. On cone C2 (Fig. 2B) fertile units are more equally sized in apical direction, suggesting that the fragment is more central in the cone. The more conspicuous fertile units and scales on the cones are discussed in the descriptions below.

**Figure 5.**
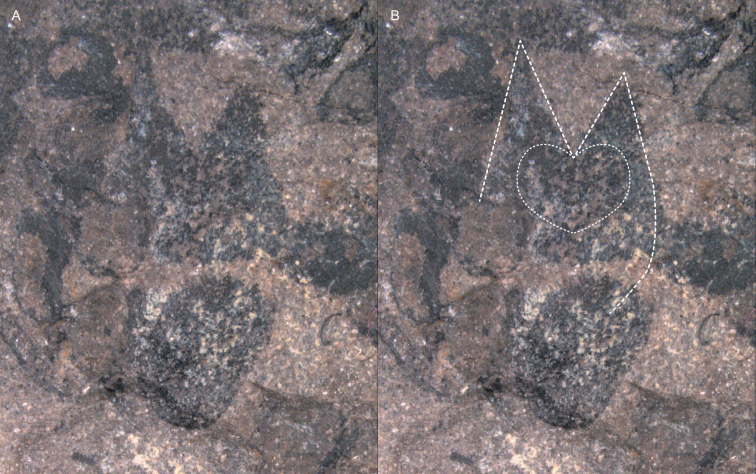
Details from Cone C1: A Facial view of the scale in the cone from sample 45311 Aa (see frame in Figure 2A) B Contours of Facial view of scale and seeds indicated by dashed lines.

A fertile scale complex occurs in facial view (Figs 2A and 5) central-basally in cone C1 on sample 45311 Aa. This scale was 3 mm wide and 5 mm long, broad at its base, bifid and elongated. The two apices are bluntly acute and not fully symmetrical. Centrally on the scale is a darker, heart-shaped structure. Apically on the left side of cone C1 on sample 45311Aa, a 2 to 3 mm scale could be observed in side view on the cone-edge (see arrow 1, Fig. 2A). Below this point on cone C1, three scales can be observed along the left cone edge, each with their apices pointing towards the cone apex. At approximately one third of the cone base of C1 on sample 45311Aa, on the left side, a larger scale in side view carries two adaxial fertile units (see arrow 4, Fig. 2A). Basally to the left side of the cone, a dark oval area is observed and considered to represent the cone axis (see arrow 5, Fig. 2A). On the right cone side (sample 45311Aa), no scales apices can be seen to protrude, but four fertile unit pairs can be observed; a thin rim abaxially to the third pair from the top indicates these fertile units are subtended by a scale. Throughout the cone, paired rounded units can be observed, indicating the cone is densely packed with seed scale complexes.

The second incomplete cone C2 on sample 45311 Aa left of the central C1 cone described above, consists of six equally sized overlapping scales (Fig. 2B). No gradual size change was observed for these fertile scales either in basal or apical direction. Here, a bifid scale is adaxially covered by another one that carries paired fertile units. This indicates that the rock split from within the cone in such a way that the fertile units are viewed from the axis and are generally positioned adaxially on top of the scales. Abaxially to the central fertile unit pairs on the right side of the cone is a scale with two long apices; abaxially from this scale is a thin rim possibly representing a subtending scale (Fig. 2A, see arrows).

The third and last, smallest incomplete cone C3 on 45311 B, is an apical fragment where three bifid scales could be recognized (Fig. 2C). Here, the rock split in such a way as to preserve the external contours of the strobilus. This cone is 6 mm wide and 10 mm in length, and the scales are approximately 3 mm wide and 3 mm long. The cone width decreases apically. Most visible scales are bifid and no convincing fertile units were observed.

### Fertile units

**Figure 6.**
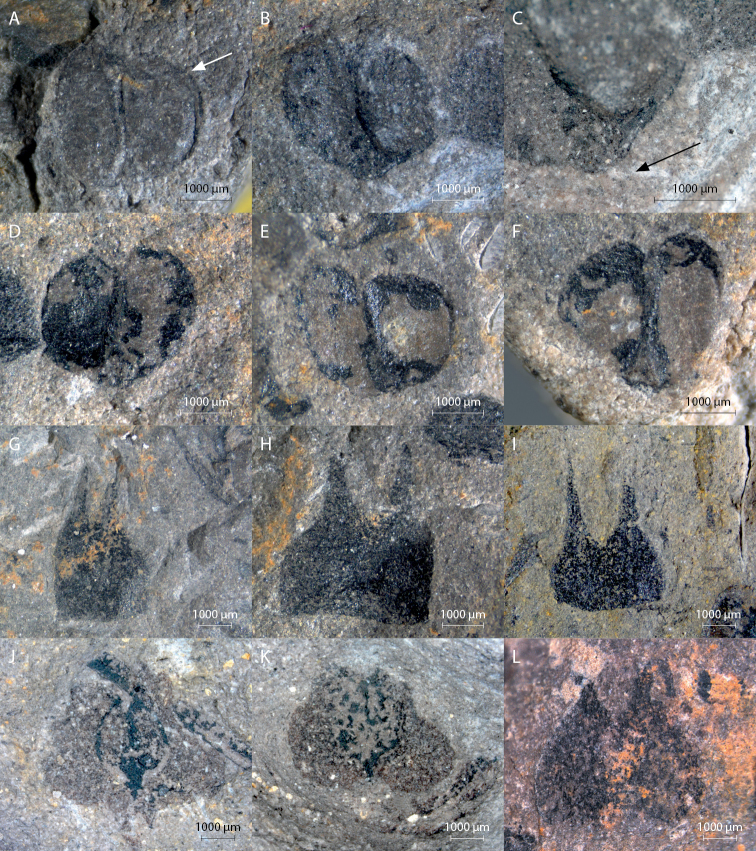
Details of dispersed seeds, scales and fertile scales: **A** paired seeds with double wall (sample 45311C) **B** paired seeds with triangular micropylar protrusion (sample 45311 E) **C** detail of triangular micropylar protrusion from paired seeds (sample 45311 E) **D** heart shaped paired seeds/ovules (sample 45311 D) **E** heart shaped paired seeds/ovules (sample 45311 B) **F** juxtaposed almond shaped seeds/ovules (sample 45311 B) **G** bicuspid scale (sample 45311 Aa) **H** bicuspid scale (sample 45311D) **I** bicuspid scale (sample 45471) **J** bicuspid scale with contour of two seeds/ovules (sample 45315) **K** bicuspid scale with darker organic contour of seed (sample 45310) **L** bicuspid scale with heart shaped contour of seeds/ovules (sample 45471).

The paired fertile units showed a variety of transitional forms from two closely adpressed hemispheres (Fig. 6A, B), to two almond – and (as a pair) heart-shapes attached both centrally and in the narrowest point (Fig. 6D, E, F). For various smaller paired fertile units there are two hemispheres, which display two very thin darker walls (see Fig. 6A). In various cases the smaller attached side of the seed pair displays a smaller triangular micropylar protrusion (Fig. 6C). Fertile units vary in length between 0.6 and 2.6 mm, and in width between 0.4 and 1.6 mm.

### Scales

The scales are bicuspid (Fig. 6G, H, I). The apices are either short (possibly broken or hidden by the rock) or can be up to half of the total length of the scales. The scales have a broadly rounded to slightly cordate base, uncommonly with a small constriction or a stalk. Length of the scales varies between 1.4 and 7.8 mm, and the width varies between 1.8 and 8.7 mm. The small constriction at the base can be 0.5 to 1.0 mm long. Scales sometimes display a striation (Fig. 3C). They can be empty or carry paired fertile units positioned centrally on the scale. Along the cone C1 on sample 45311Aa, scales all have approximately the same length.

### Fertile unit pairs on scales

On the scales the pair of fertile units forms an oval consisting of two hemispheres or a heart-shape pointing adaxially on the scale (Fig. 5, Fig. 6J, K, L), sometimes sprouting from the scale stalk. Other fertile units form two oval shapes attached centrally to the scale.

### Quantitative analysis

The search for measurable organs resulted in 263 fertile units and 158 scales. Fertile units were observed in the cones, on scales or dispersed; scales were observed in the cones, dispersed fertile or empty (Suppl. materials 1, 2).

### Fertile unit, length, width and size proxy (n=263)

**Figure 7.**
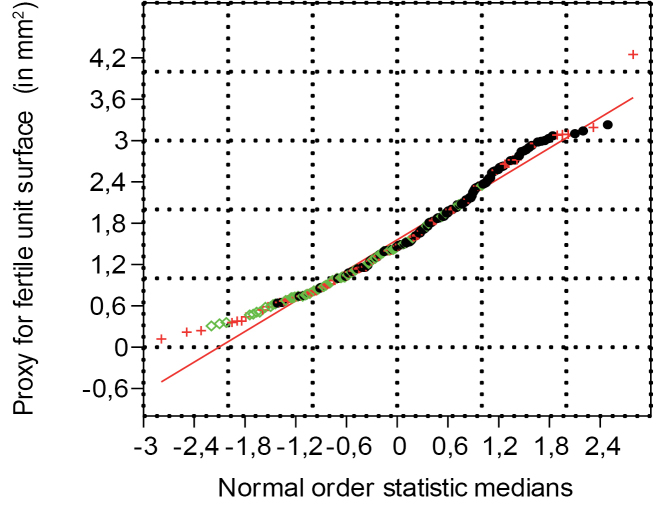
Normal probability plot for the seed surface (length x width) proxy (n=263) (green represents seeds in cone, red represents seeds on scales, and black represents dispersed paired seeds).

The set of 263 data points for the fertile unit length and width have a correlation coefficient of, respectively, 0.9976 and 0.9966 from the normal probability plot (Fig. 7). At the 5% significance level, the critical value for 260 to 270 data points varies between 0.9945 and 0.9947. Since 0.9976 and 0.9966 are larger than the critical values of 0.9947 and 0.9945, we cannot reject the null hypothesis that the fertile units came from a population with a normal distribution. This indicates that the isolated fertile units, the fertile units on the dispersed scales and those in the cone, all belong to the same population showing normal distribution.

**Figure 8.**
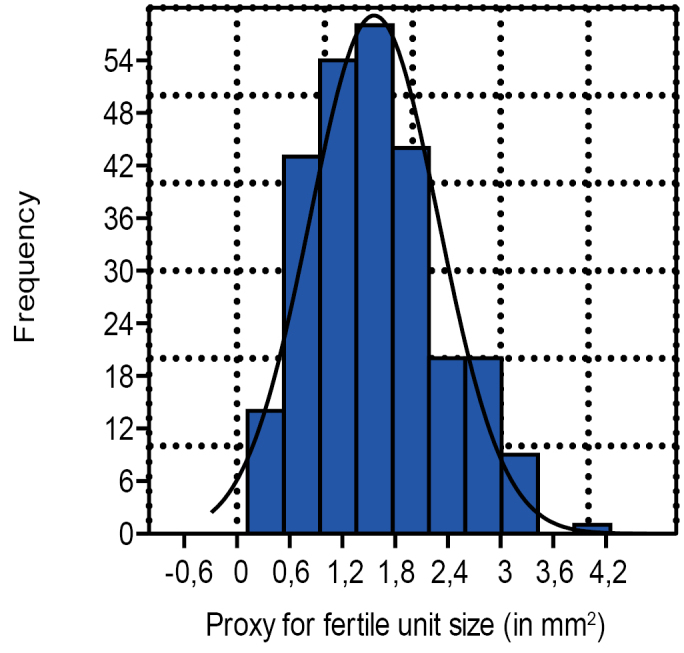
Histogram for the seed surface (length x width) proxy distribution compared to the normal distribution curve (n=263).

The histogram of the proxy for size (length x width) of all fertile units, for example, in cones, on fertile scale and dispersed (Fig. 8), indicates a slight positive skewness. Calculated in excel it is 0.54, and indicates that smaller fertile units appear slightly more commonly than larger ones.

**Figure 9.**
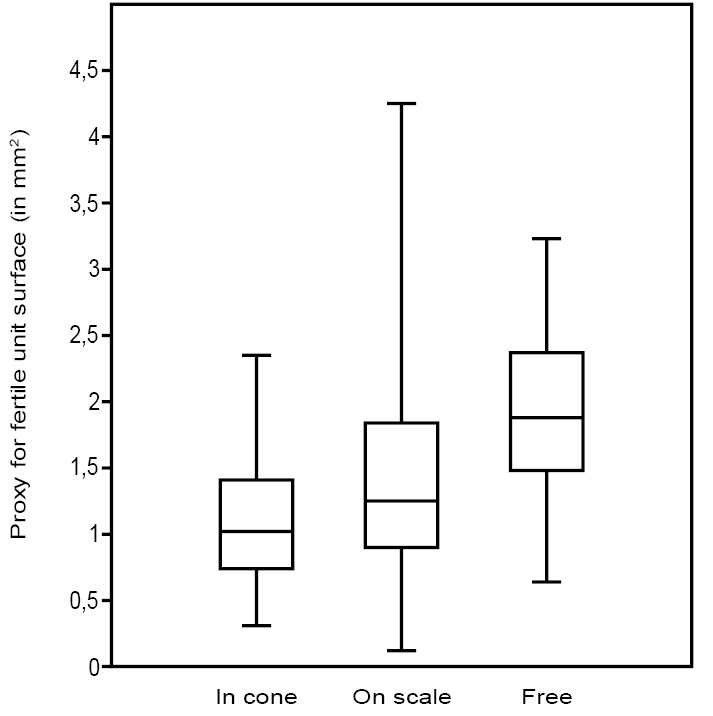
Comparison between the box charts of the proxy for seeds size (length x width) in cones, on scales and free.

The box charts of the fertile unit size proxies (Fig. 9) indicate that, on average, the fertile units in the cones are smallest and the dispersed fertile units without the scale are the largest. Fertile units on the scales have an average size, but their size range is the largest.

**Figure 10.**
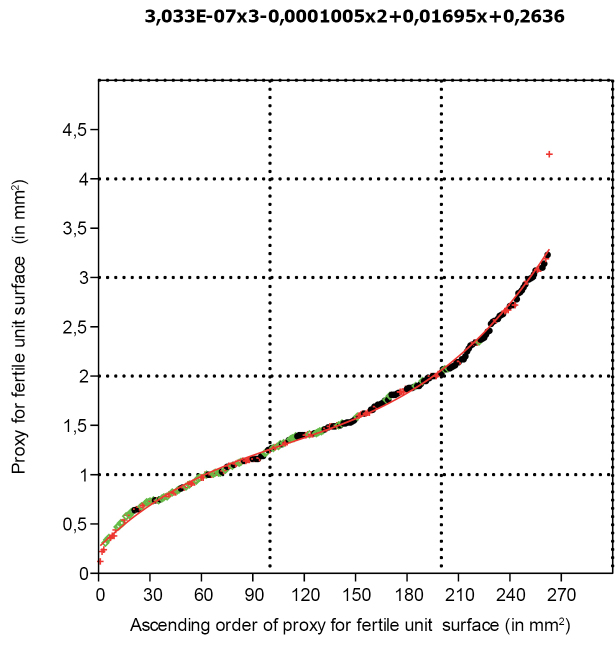
Proxy for seed surface (length x width) organised in ascending order with the superimposed polynomial function describing it (n=263).

The distribution of the proxy for the size of the fertile units ranked in ascending order can be described with a polynomial function of the third order. In the lower part of the spectrum, fertile units are chiefly positioned in cones. In the central part, fertile units can be positioned either in cones, on dispersed scales or are free, while in the upper part of the spectrum fertile units are either free or on dispersed scales, but more commonly free (Fig. 10).

### Scale length, width and size proxy (n = 158)

**Figure 11.**
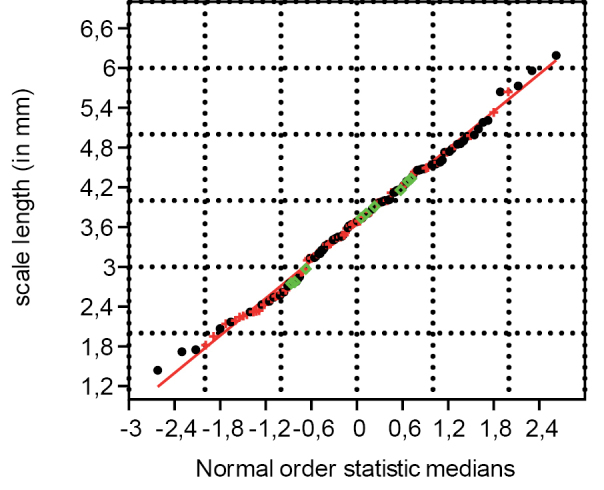
Normal probability plot for the scale length (n=158). Green represents scales in cone, red represents dispersed fertile scales, and black represents dispersed empty scales.

**Figure 12.**
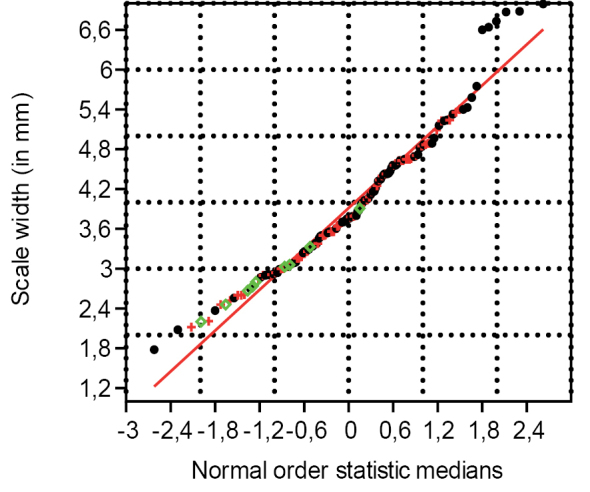
Normal probability plot for the scale width (n=158) (green represents scales in cone, red represents dispersed fertile scales, and black represents dispersed empty scales).

The set of 158 data points for the scale length and width have a correlation coefficient of, respectively, 0.9975 and 0.9821 from the normal probability plot (Figs 11, 12). At the 5% significance level, the critical value for 150 to 160 data points varies between 0.9909 and 0.9915. Since 0.9975 for the scale length is greater than their respective critical values of 0.9909 and 0.9915, we cannot reject the null hypothesis that the scales came from a population with a normal distribution. This indicates that the length of empty scales, fertile scales and scales in cones belong to the same normally distributed population. As the width of the scales has a high PPCC, yet lower than the critical value, the width of the scales is not normally distributed. When taking the four widest scales out of the analysis, the PPCC is higher than the critical value, indicating that most scale widths are normally distributed and, consequently, most scales, in cones, fertile scales and empty dispersed scales, belong to the same normally distributed population with respect to their width.

**Figure 13.**
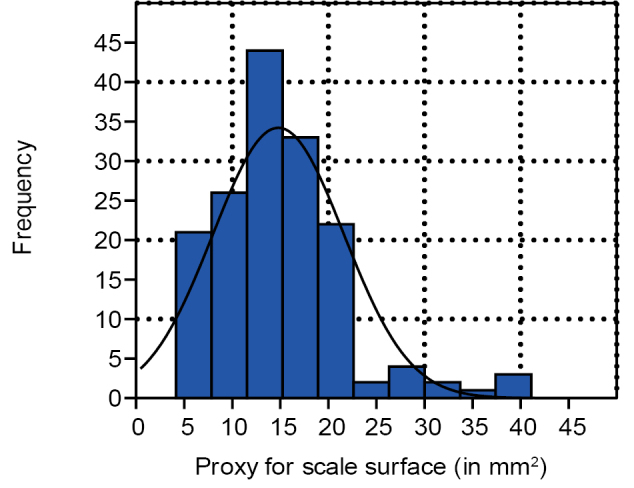
Histogram for the proxy for scale surface (length x width) distribution compared to the normal distribution curve (n=158).

The histogram of the proxy for size of all scales – for example, dispersed either empty or fertile – and in cones (Fig. 13), compared to the normal distribution curve, indicates a positive skewness for the fertile unit surface proxy, yet more pronounced; in Excel it has a value of 1.3. It indicates that smaller scales are more common than larger ones.

**Figure 14.**
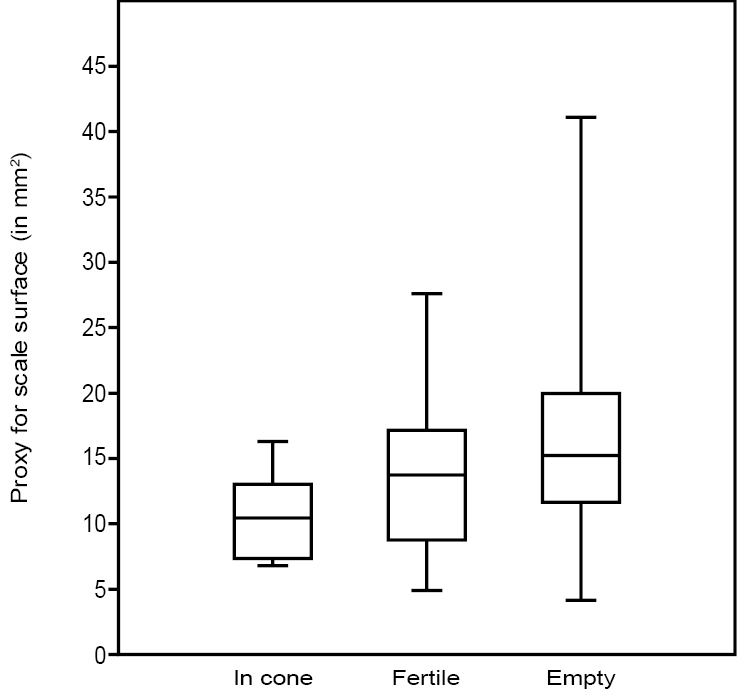
Comparison between the box charts of the proxy for seeds surface (length x width) in cones and dispersed, fertile and empty (n=158).

The box charts of the scale size proxies (Fig. 14) indicate that, on average, the scales in the cones are smallest while the dispersed empty scales are largest. The size of fertile scales is in between the two.

**Figure 15.**
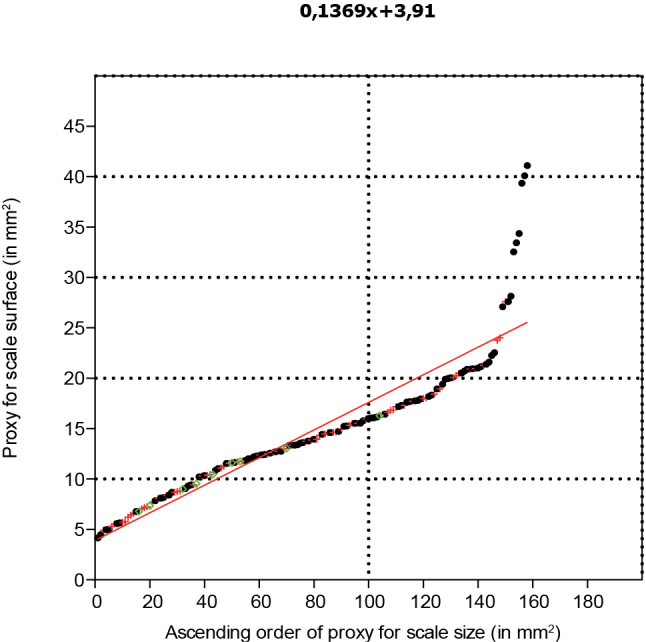
Proxy for scale surface (length x width) organised in ascending order (n=158).

The distribution of the proxy for the size of the scales (Fig. 15), ranked in ascending order, diverges from the polynomial function depicted above for the proxy distribution of the fertile units size. It can best be described as three linear fragments: an initial segment with small scales; a second fragment with a lower angle with medium sized scales; and a third steep segment with the largest scales. This first segment holds scales from all three categories, that is, in cones, fertile and empty; the second segment as well, but scales from cones are less common; and the third segment holds but two fertile scales, while all others are empty scales. The 15 widest, empty scales all fit in one linear segment also comprising two fertile scales. The segment itself has a PPCC of 0.9619, well above the critical value for 15 specimens of 0.9376, indicating the segment itself has a normal distribution and that each specimen from this segment (including the fertile specimen) belongs to the same normally distributed population. As fertile scales are by definition what Jongmans and Gothan (1925, 1935) considered *Tobleriabicuspis*, scales from this last normally distributed linear segment are also considered to belong to *T.bicuspis* with confidence.

### Results of fluorescence analysis

**Figure 16.**
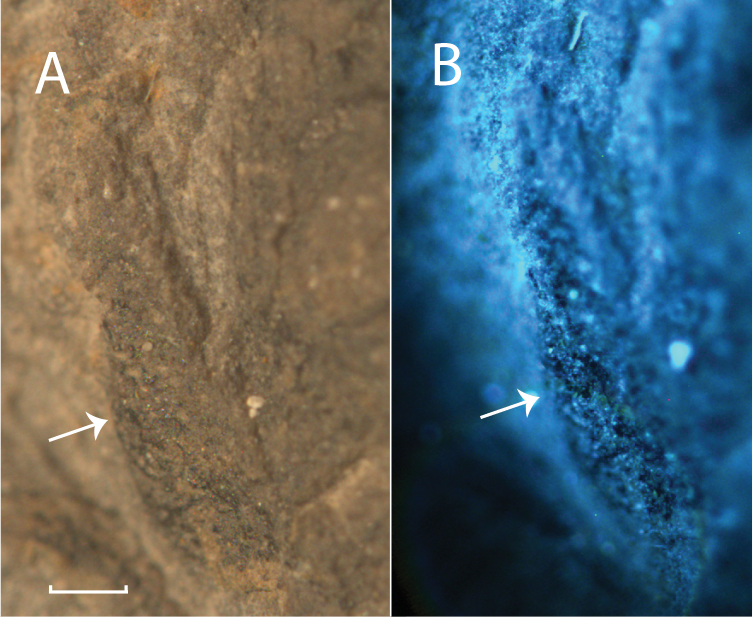
Absence of fluorescence in a scale from the Cone C1 from 45311 Aa: **A** scale under normal light showing the scale as being slightly darker than the rock **B** identical scale with fluorescent light showing the scale as being opaque while the rock around it shows some fluorescence (arrows indicate the scale).

The scales and seeds, both dispersed and in the cone, were exposed to fluorescent light. While the sedimentary rocks holding the plant fossils displayed fluorescence, the scales and seeds were totally inert (Fig. 16A, B, see arrows).

## Interpretation and discussion

### Seed cones

The statistical analysis conducted above indicates that the paired fertile units and bifid scales belong to one population also represented in the cones. From the box charts it appears that the larger they are, the more the fertile units are dehisced. The fertile units are initially attached to the cone and then dispersed still on a scale to final dispersal without the scale. Considering size generally as a measure for maturity, it appears that more mature fertile units are more easily released. This relation is interpreted here as a natural shedding strategy and as such is applicable to an ontogenetic development from ovules to ripened seeds. The present box charts, indicating a gradual release of the fertile units, supports such an interpretation as seeds. This is often seen in younger cones; numerous Mesozoic seed cones have been found falling apart, and in *Cedrus* and *Abies* the seeds become loose as the cone matures (Harris 1976). Jongmans and Gothan (1925, 1935) also considered *Tobleriabicuspis* as ‘seeds’ on scales rather than sporophylls bearing microsporangia because they found no pollen grains in their palynological preparations.

Two thin walls were observed around the smaller dispersed fertile units (Fig. 6A, see arrow). As some ovules display a small, darker, cone-like protrusion interpreted here as a micropyle enclosing a small pollen chamber, the outer wall is considered as the integument and the inner wall as the nucellus. The micropylar protrusions were observed on the smaller side of the heart-shaped paired seeds (Fig. 6C, see arrow) which were pointing towards the base of the scale (see Fig. 5A, with interpretation in B); the ovule is consequently interpreted as inverted. No wing was observed on the seeds. *Tobleriabicuspis* is, in consequence, interpreted as a compact cone composed of helicoidally-placed, paired anatropous ovules/seeds sessile on a bifid scale.

### Taxonomic affinity

Seed cones from the Carboniferous-Permian transition have either a cycadalean, a gnetalean or a peltaspermalean affinity; or, within the conifers, may belong to the Cordaitanthales, the Ferugliocladales, the Dicranophyllales or the Voltziales. Early Permian cycadalean fertile scales differ from those of *T.bicuspis* as their seeds are positioned laterally to the sporophyll base (Gao and Thomas 1989). The gnetalean cones from the late Permian of China (Wang 2004) differ from those of *T.bicuspis* as they have a lanceolate bract and a single megaspore adnate to the bract. Peltaspermales are, as their name indicates, peltate, which is not the case in *Tobleria*. Cordaitanthalean seed cones differ from those of *Tobleriabicuspis* because entire scales subtend shoots with erect ovules form a seed with a conspicuous wing (Ignatiev and Meyen 1989). Early Permian Ferugliocladales seed cones are characterized by an orthotropous ovule attached directly to the cone axis while *T.bicuspis* has sessile anatropous ovules. Cheirolepidiales cones have trifid scales encapsulating a single seed, while the bract is entire (Anderson et al. 2007).

Cones with paired anatropous ovules occur in the voltzian conifers. Most typically the seeds/ovules of *Tobleriabicuspis* are sessile on the scale, similar to the informal group coined by Rothwell et al. (2005) of the voltzian Voltziales, like, for instance, *Majonica*, *Dolomitia*, *Ullmannia*, *Voltzia*, *Pseudovoltzia*, *Voltziopsis and Schizolepis*, amongst others. They are not carried directly by the fertile shoot axis as is the case in the informally coined group of walchian Voltziales (Rothwell et al. 2005) like in *Emporia* or *Ernestiodendron. Tobleriabicuspis* consequently is most reminiscent of a voltzian Voltziales.

Dicranophyllales also represent early conifers with paired seeds on a helicoidally-placed scale in a compact cone, but the seeds are erect and winged (Noll 2011). In Rothwell et al. (2005), *Dicranophyllumhallei* still appears as transitional between the Cheirolepidiales and the Voltziales, but would, considering the latest description (Noll 2011), rather fit in the Voltziales. As the seeds in *T.bicuspis* are inverted and carried by a scale, they have characters of the voltzian Voltziales.

### Bract adnate, closely packed or absent

Voltzian Voltziales are generally described with a bract, but there is little evidence available for *T.bicuspis* to establish the presence of a bract subtending the scale. There are more dispersed empty scales than dispersed paired seeds, thus indicating that all paired seeds are accounted for by a scale while only 55% of the dispersed paired seeds are accounted for by a second empty scale that can be hypothesized to represent a bract. Various solutions can be offered for the discrepancy between the proportion of fertile and empty scales. A taphonomic rationale can explain this bias by considering that scales are relatively lighter than seeds and that they are more easily transported, leaving relatively more seeds behind. Another solution resides in the fact that the fertile scales and bracts can be expected to be adnate or closely packed; therefore, it is difficult to distinguish one from the other in the dispersed material.

The Voltziales*Schizolepismoelleri* and *S.planidigesita* from the Middle Jurassic of the Liaoning (Wang et al. 1997) have been figured with a bifid bract subtending an equally bifid scale. Scales and bracts having a comparable shape masking their function can consequently be expected in the Voltziales.

*Schizolepispermensis*, from the Kupferschiefer of Funfkirchen, based on one scale only with a pair of inverted seeds, was described without a bract (Heer 1876); this is not an isolated case as the same is the case for the early Cretaceous *Schizolepislongipetiolus* of which the cone is reconstructed without bracts (Xu et al. 2013), while in Zhang et al. (2011, table 1) 17 out of 23 listed *Schizolepis* species are without bract description. Moreover, various *Schizolepis* species have reduced bracts (Wang et al. 1997) suggesting that bract reduction may also have led to bract absence or to difficulties in observing the bract. The absence of a bract is consequently a common feature for various *Schizolepis* species. *Schizolepis* has been placed in the Voltziaceae (Schweitzer and Kirchner 1996, Anderson et al. 2007); Voltziaceae are typified by a fertile scale, rather than a fertile shoot, and belong consequently to the informal group coined by Rothwell et al. (2005) as the voltzian Voltziales.

### Comparison to Schizolepis

Comparably to *Schizolepis* species, *Tobleriabicuspis* is characterized by deeply incised scales (Schweitzer and Kirchner 1996, Wang et al. 1997, Heer 1876, Zhang et al. 2011, Xu et al. 2013). In this respect *T.bicuspis* differs from *Gomphostrobus* (Marion 1890) which has an apically bifurcating scale. It also contrasts with the bracts of *Emporia*, *Ernestiodendron*, *Otovicia* and *Barthelia* (Anderson et al. 2007), all of which display a minor apical bifurcation.

Consider the suite of features shown by *Tobleriabicuspis*: an apically compact cone composed of helicoidally-placed scales, seeds being shed, ovules with a double wall and a small micropyle, scales with adaxially paired anatropous sessile ovules, deeply incised scales, and the absence of a seed wing. These features indicate that *Tobleriabicuspis*, in spite of the difficulties in unequivocally demonstrating the presence of bracts, mostly resembles the voltzian Voltziales and is reminiscent of *Schizolepis*, but differs from it in that the latter’s cones are generally lax (Wang et al. 1997, Zhang et al. 2011).

The resemblance to *Schizolepis* is remarkable as it is commonly found in Mesozoic strata (Schweitzer and Kirchner 1996, Zhang et al. 2011, Xu et al. 2013, Wang et al. 1997) and is often considered as a Pinaceae (Krassilov 1982, Wang et al. 1997, Zhang et al. 2011) because of the discovery of a wing to its seed (Wang et al. 1997, Zhang et al. 2011). It should be mentioned that this pinaceous affinity is disputed by some (Anderson et al. 2007) or found to be very distant (Xu et al. 2013).

### An early voltzian Voltziales in an early extrabasinal palaeoflora?

An evolutionary development from a radial ovuliferous shoot to an ovuliferous scale is a common concept for the origin of the voltzian Voltziales (Meyen 1997, Anderson et al. 2007, Taylor et al. 2009). Taxa with an ovuliferous shoot in a leaf axil are found in late Pennsylvanian strata (Anderson et al. 2007), while derived Voltziales with only one scale and sessile seed like the Ullmanniaceae are described from the early Permian (Roadian) Kupferschiefer (Schweitzer 1963). Transitional taxa with paired seeds and bifid scales can consequently be expected between the Pennsylvanian and the Roadian. *Tobleriabicuspis* represents such a transitional taxon and is found in the Mengkarang Formation. The Mengkarang Formation is late Asselian, for which an isotopic age was measured between 296.77 and 296.14 million years (Van Waveren et al. 2018). *Lebowskia*, the earliest voltzian Voltziales described until now, was found in the slightly younger strata of the late early Permian Leonardian (270 to 280 million years old) (Looy 2007). *Tobleriabicuspis* can consequently be seen to represent a voltzian Voltziales occurring 16 to 26 million years earlier than was described until now. *Dicranophyllumhallei* from the 290.7 million years old Donnersberg Formation (Lippolt and Hess 1989) represents a voltzian Voltziales, as seen above, only 6 million years younger than *Tobleriabicuspis*.

The early occurrence of *Tobleriabicuspis* is not as surprising as it may seem as it was found in the Mengkarang Formation where gravity flows from the extrabasinal volcanic slope environment are characterized by very early seed fern occurrences (Booi et al. 2008, 2009a, 2009b). Earliest American gigantopterids (Koll et al. 2017), for instance, are 12 million years younger than *Gothanopteris*, a gigantopterid from the Mengkarang Formation gravity flows. *Tobleriabicuspis*, consequently, is part of the extrabasinal environment where Rothwell et al. (2005) expected greater evolutionary innovations in conifers. It illustrates, in particular, the traditional view that stressed environments, such as those of the volcanic slopes from the Karing Volcanic Complex (Van Waveren et al. 2018) must have been, are especially prone to morphological novelties.

### Late Palaeozoic conifer diversity

Voltziales with ovuliferous shoots and those with only ovuliferous scales are not necessarily mutually exclusive. *Molyostrobustexanum* from the early Permian of Texas has a single erect ovule carried by a flattened shoot (Miller and Brown 1973), while the paired seeds in the Sakmarian *Dicranophyllumhallei* are carried by a scale (Noll 2011). This indicates that Voltzialean conifers with shoots and scaled Voltzialeans co-existed at the Permo-Carboniferous transition. It has even been suggested that co-occurrence of Palaeozoic coniferophytes, for example, *Pseudovoltzia*, with a complete reduction of the dwarf shoot with others like *Buriadia* or *Ferugliocladus* with stalked ovules attached directly to the cone axis (Archangelsky and Cuneo 1987), may indicate that there were two or more distinct lineages present at this time (Serbet et al. 2010). Considering the diversity of coniferophytes from the late Palaeozoic, the assumption of Florin (1951) that they all can be traced to a single ancestor is, perhaps, debatable.

### Early accidental cone shedding or matured cone?

The positive skewness in the histogram for seeds and scales size proxies in *Tobleriabicuspis* indicates that smaller fertile units and scales occur more commonly than larger ones. Fossils cones, in general, can be represented by numerous separate scales, while accidentally detached cones, on the contrary, still can hold seeds and scales (Harris 1976). In the case of *T.bicuspis*, the dominance of smaller seeds and scales can be explained by accidental early cone shedding arresting all developments. Accidentally shed cones are expected to be intact (Harris 1976), which is not the case for *Tobleriabicuspis* that partly fell apart as has been demonstrated by the statistical study conducted above. On the other hand, the size distribution of ovules or seeds may also relate to poor fertilisation success in a fully grown cone. In the absence of size studies of seeds in conifers, comparison is drawn here with extant Zamiaceae where the ovule/seed size is represented by three categories: the ovules that have not been pollinated which are smallest, the pollinated ovules and abortive seeds that are medium-sized, and the mature seeds that are largest (Mora et al. 2013). In the fertile unit size proxy distribution (Fig. 10), three diagram segments appear, a segment with small fertile units, a segment with middle-sized fertile units and a steep segment with largest fertile units. These three segments can be hypothesized as indicating that the smallest fertile units are not pollinated, the medium-sized are aborted or pollinated but not matured, while the last segment represents matured seeds. For *Dioonedule* seed efficiency was only 42.5% which was considered to indicate that major seed loss was attributable to ineffective pollination (Mora et al. 2013). The positive skewness of *T.bicuspis* seeds and scales consequently may indicate either that cones were shed while being immature, or that there was ineffective pollination, or a mixture of both. Considering the low number of large seeds, cone shedding while the cone was chiefly immature seems to have been the case for *Tobleriabicuspis*.

In extant taxa, *Cedrus* and *Abies* for example, scales and seeds disperse separately when the cone ripens while in *Araucaria* the seed, enclosed in the cone scale, disperses as one unit (Harris 1976). The relation between size and the degree of release of seeds/ovules as described for *T.bicuspis* above suggests both seed shedding strategies, on scales or separate seeds and scales, operate simultaneously, while the latter dominates because the fertile scales are fewer.

### Reconstruction of the *Tobleriabicuspis* cone

The scale size proxy curve is also represented by three segments with three different steepnesses, but these are clearly linear: (1) small scales with a steep size distribution; (2) medium-sized scales with a moderately steep size distribution; and (3) large scales with a steep size distribution. Considering growth to be apical, the largest scales are interpreted as representing the scales near the cone base. As these large scales are chiefly empty and because a steep diagram fragment was also observed for the fertile unit size distribution, indicating that the largest paired seeds were often dehisced from the scale (Fig. 10), these larger empty scales are hypothesized to have shed their seeds. Because both the large paired seeds and the large scales belong to a steep segment of their size distributions, it is hypothesised to represent a zone of rapid maturation.

**Figure 17.**
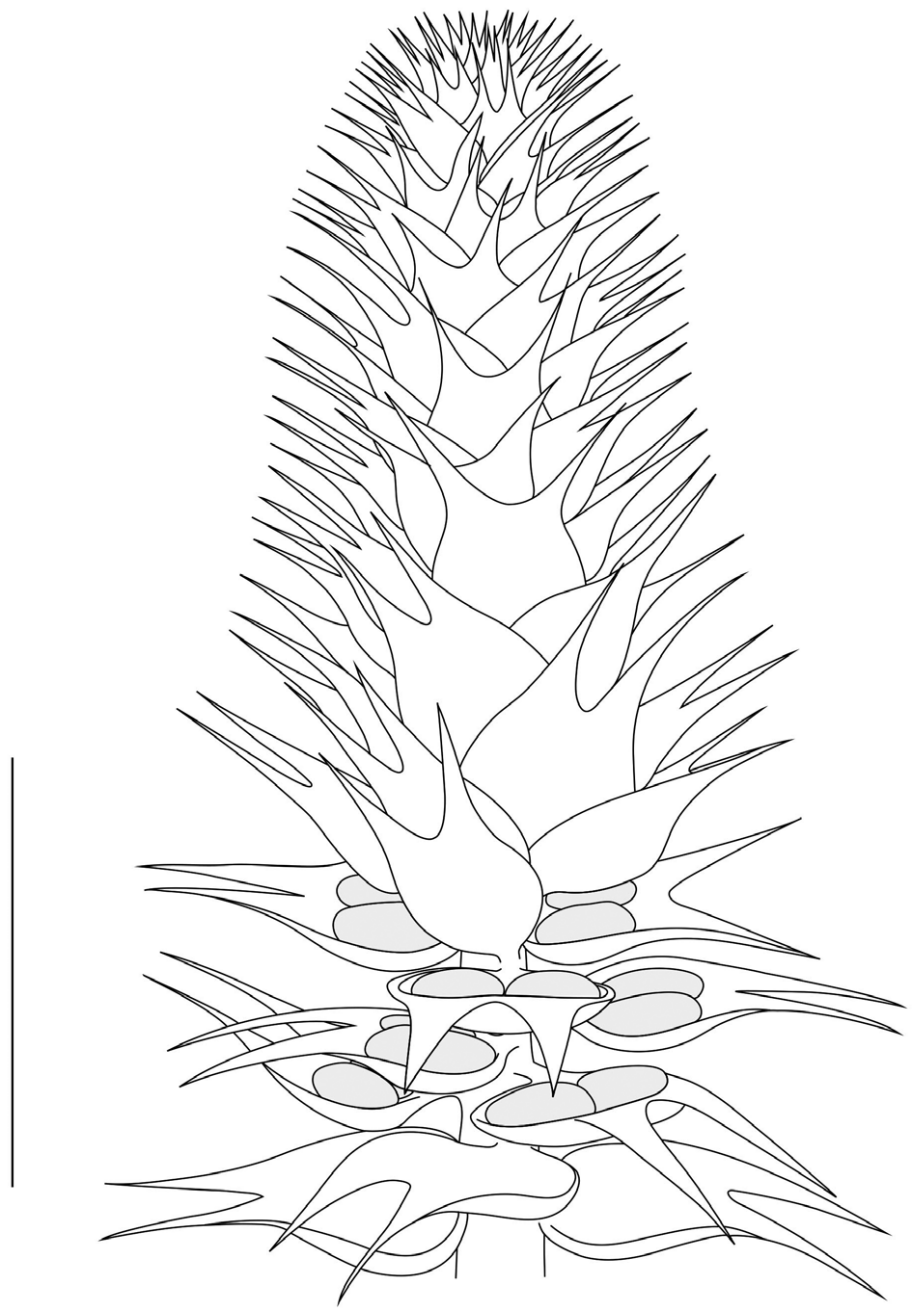
Reconstruction of *Tobleriabicuspis*. Scale bar: 1 cm.

The second segment of the scale size distribution comprising empty scales, fertile scales and scales in the cones is expected to represent the central part of the cone. As the largest scales still attached to the cones are only found in the lower half of the second segment of the scale size proxy distribution, cones are expected to be twice the length of the longest fossil cone fragment found in the Jambi collection. The first segment with the smallest ones comprises most scales found in the cones and is expected to chiefly represent its apical part, last developed. Such smaller scales, when being dispersed, may also represent a constricted cone base, but there is insufficient evidence to allow for its reconstruction. In consequence, the reconstruction presented here does not comprise the very base of the cone, but a zone of large empty scales hypothesized to represent fertile scales that have shed their seeds (Fig. 17).

In some cones of *Compsostrobus*, there is a fertile zone where the scales and bracts are attached at a less acute angle, a feature that is considered to be associated with maturation (Delevoryas and Hope 1973). Because of the sudden and steep positive excursion of the proxy for scale size at the high end of its distribution, and because of the presence of relatively large dispersed ones in the fertile unit distribution, it is suggested here that such a zone of rapid maturation was also found in *T.bicuspis*. This suggestion adds an explanation for the positive skewness of the fertile unit size proxy distribution, as it is speculated that dominance of smaller fertile units follows from functional protection from pollination and ripening due to a more acute positioning of the scales for most of the cone.

### Associated palaeoflora

*Tobleria* seeds were found in association with the mesic-xeric elements from the Jambi palaeoflora. Gymnosperm leaves from these localities are *Cordaitesprincipalis*, *Macralethopterishallei*, *Sphenopteris* sp., *Dicranophyllummolle* and Peltaspermales. *Gothanopterisboschana* and taeniopterids of which the taxonomic affinities are unknown, are also part of this mesic-xeric association from the Mengkarang Formation (Booi et al. 2008, 2009a, 2009b). *Dicranophyllummolle* is a leaf species found on specimens holding *Tobleriabicuspis* scales and seeds (Van Waveren et al. 2007). Considering that the early conifer *Dicranophyllumhallei* with a comparable leaf habit (Barthel et al. 1998) carries cones on its axis, *Dicranophyllummolle* appears to be a good model for the vegetative form of *Tobleriabicuspis*.

### Associated pollen and spores

Palynological analysis of only one sample from the base of the Mengkarang Formation gives good results (Crippa et al. 2014). The sample is dominated by lycopsids like *Laevigatosporites* spp., indeterminate bisaccate pollen representing a broad range of gymnospermous taxa, *Florinites* representing the cordaites and *Convolutispora* sp. representing ferns. The assemblage also contains very small numbers of other taxa of which *Alisporites* spp., ?*Divarisaccus* sp., *Circumstriatites* spp. and Protohaploxypinuscf.limpidus represent gymnosperms, while the remaining *Punctatisporites* spp., *Raistrickia* sp., *Calamospora* spp. and *Sulcatisporitesovatus* represent spore forming plants. *Tobleriabicuspis* has a robust axis and it is considered to represent an erect cone with, as said above, inverted ovules. According to Leslie (2008), such erect cones with inverted ovules are likely to have been pollinated by bisaccate pollen, leaving a broad range of possibilities within the palynological sample described above for the pollinator of *Tobleriabicuspis*. It should be stressed that, in contrast to the palynological sample described above, *T.bicuspis* was found in the upper half of the Mengkarang Formation during which time, according to Van Waveren et al. (2018), ecological circumstances were more xeric. Indeed, *T.bicuspis* was found in strata at least half a million years later than the time of origin of the palynological sample and it is questionable if *Tobleriabicuspis* was already part of an upland palaeoflora during the climatic circumstances from the base of the section which were depicted by Van Waveren et al. (2018) as tropical wet.

### Palaeoecological implication of the taphonomy of the samples

The *Tobleriabicuspis* seeds and scales that are from the Ketidoeran Siamang River locality, representing the upper half of the Mengkarang Formation, are considered to have originated from the source area of the gravity flows and are consequently allochtonous. The cones from the Karing River, at the top of the Mengkarang Formation, have not been transported significantly and are considered to be parauchtonous. This indicates that *Tobleriabicuspis* grew on the slope of the Karing Volcanic Complex during what was indicated by Van Waveren et al. (2018) as the falling stage of a third order eustatic sea level fluctuation, while during the low stand *T.bicuspis* grew at the foot of the volcano. This palaeoecological habitat transition can be hypothesized as being climatically induced as mesic-xeric taxa, occupying the volcanic slope during periods of climate deterioration, appeared to move to wetlands at the base of the volcanic slope when climate deteriorated.

### Petrography and fluorescence

The *Tobleriabicuspis* samples were not exposed to petrographic analysis, unlike 33 samples from the Merangin section through the Mengkarang Formation. Ten out of these 33 organic-rich samples clearly contain leaf remains and indicate between 36 and 89% of inertinite, the remaining percentages being vitrinite and in four cases also low ratios of liptinite. The inertinite in these samples was chiefly composed of fusinite, but also semi-fusinite, macrinite, detrinite and inertodetrinite (Sýkorová and Šulc 2009). As none of the 33 samples subjected to petrographic analysis is composed for a 100% of inertinite (fusinite in particular), forest fire, considered to form inertinite and fusain (Scott 2000), probably played little to no part in the preservation of the Jambi palaeoflora.

On the other hand, the seeds, scales and cones of *Tobleriabicuspis* displayed no fluorescence at all. Inertinite, as opposed to vitrinite and leptinite, displays no fluorescence (Tyson 1995); thus, the *T.bicuspis* remains are inertinite. The detailed morphology even suggests they are composed of a particular category of inertinite, namely fusinite. The early appearance of voltzian Voltziales in the Asselian can therefore not only be explained by a unique depositional setting giving a rare window into the palaeoecological consequences of a low stand within an icehouse period in the tropics (Van Waveren et al. 2018), but also by the outstanding preservation circumstances that follow from the formation of inertinite or fusinite by forest fires.

## Conclusion

*Tobleriabicuspis* is regarded as a compact cone, with helicoidal paired seeds on bifid scales. The cone dehisced (dispersed) and released fertile scales, scales and seeds. The most important aspect of the present description is the demonstration of the existence, in early Permian times, of a coniferophyte with scales, if not scale/bract complexes, with a derived voltzian Voltzialean architecture. The radial symmetry of ovuliferous shoots of the walchian Voltziales, common to the late Palaeozoic, cannot be detected. *Tobleriabicuspis* is part of the mesic-xeric Jambi palaeoflora from the West Sumatra volcanic region, where other gymnosperm taxa also appeared relatively early.

## Supplementary materials

Supplementary material 1 Measurements of 263 fertile units Data type: measurement Explanation note: Column 1: “cone” indicates the fertile units found in cones, “on scale indicates the fertile units found on dispersed scales, “free” indicates the fertile units found dispersed free from scale; column 2 gives the length of the fertile units in mm; column 3 gives the width of the fertile units in mm; column 4 gives length x width and represents a proxy for the fertile unit size. This dataset is made available under the Open Database License (http://opendatacommons.org/licenses/odbl/1.0/). The Open Database License (ODbL) is a license agreement intended to allow users to freely share, modify, and use this Dataset while maintaining this same freedom for others, provided that the original source and author(s) are credited.Isabel M. Van Waveren

Supplementary material 2 Measurements of 158 scales Data type: measurement Explanation note: Measurements of 158 scales: Column 1: “In cone” indicates the scales found in cones, “fertile dispersed” indicates the dispersed scales that are fertile, “empty dispersed” indicates the empty dispersed scales. Column 2 gives the length of the scales in mm; column 3 gives the width of the scales in mm; column 4 gives length x width and represents a proxy for the scale size. This dataset is made available under the Open Database License (http://opendatacommons.org/licenses/odbl/1.0/). The Open Database License (ODbL) is a license agreement intended to allow users to freely share, modify, and use this Dataset while maintaining this same freedom for others, provided that the original source and author(s) are credited.Isabel M. Van Waveren
